# Clinician’s Guide to Using Ozanimod for the Treatment of Ulcerative Colitis

**DOI:** 10.1093/ecco-jcc/jjad112

**Published:** 2023-07-12

**Authors:** Bruce E Sands, Stefan Schreiber, Irina Blumenstein, Michael V Chiorean, Ryan C Ungaro, David T Rubin

**Affiliations:** Dr Henry D. Janowitz Division of Gastroenterology, Icahn School of Medicine at Mount Sinai, New York, NY, USA; Department of Internal Medicine I, University Hospital Schleswig-Holstein, Kiel University, Kiel, Germany; Medical Clinic 1, Department of Gastroenterology, Hepatology and Clinical Nutrition, Goethe University Hospital, Frankfurt, Germany; Department of Gastroenterology, Swedish Medical Center, Seattle, WA, USA; Dr Henry D. Janowitz Division of Gastroenterology, Icahn School of Medicine at Mount Sinai, New York, NY, USA; Section of Gastroenterology, Hepatology & Nutrition, University of Chicago Medicine Inflammatory Bowel Disease Center, Chicago, IL, USA

**Keywords:** Ulcerative colitis, ozanimod, S1P receptor modulator

## Abstract

The emergence of advanced therapies [eg, biologics, Janus kinase inhibitors] over the past few decades has revolutionised the treatment of ulcerative colitis. However, the limitations of these therapies leave an unmet need for safer and more effective or convenient treatment options. There is growing interest in the development of novel oral small molecule therapies for the treatment of ulcerative colitis. Ozanimod is an oral small molecule therapy that is approved in the USA, the European Union, and other countries as the first sphingosine 1-phosphate receptor modulator for the treatment of moderately to severely active ulcerative colitis in adults. This review provides guidance for ozanimod use for the treatment of ulcerative colitis, based on the prescribing information, clinical trial and real-world data, and the authors’ clinical experiences. This guidance outlines patient characteristics to consider when deciding if ozanimod treatment is suitable and describes how to educate patients on risks and best practices. It also details the nature and frequency of monitoring during treatment, which should be adapted to the individual patient based on pre-existing risk factors and events that possibly occur during treatment. This review also provides insights into the patient characteristics and clinical scenarios best suited for ozanimod treatment, based on its efficacy, safety profile, and risks compared with other therapies.

## Graphical Abstract

**Graphical Abstract:**
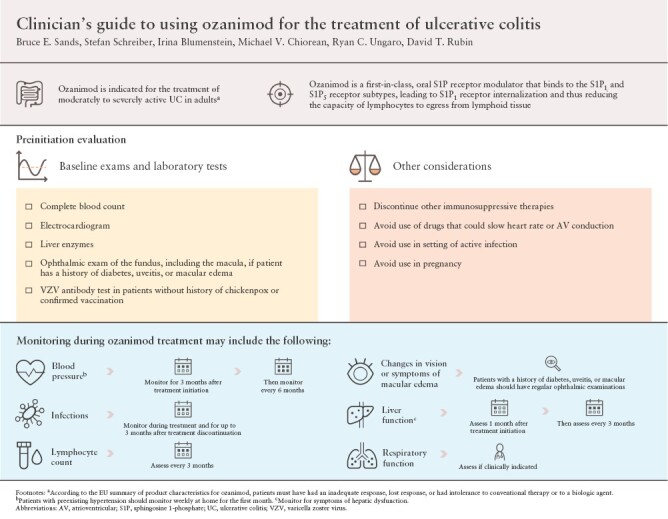

## 1. Introduction

Ulcerative colitis [UC] is a chronic, relapsing-remitting, inflammatory bowel disease [IBD] characterised by diffuse mucosal inflammation of the colon.^1^ The incidence and prevalence of UC have increased over the past 50 years and continue to rise globally.^2–4^ Conventional treatments for UC include aminosalicylates, corticosteroids, and thiopurines [eg, azathioprine, 6-mercaptopurine].^5^ The emergence of biologic agents (ie, tumour necrosis factor [TNF] inhibitors, integrin inhibitors, and interleukin 12/23 inhibitors) has revolutionised the treatment of moderately to severely active UC over the past two decades,^5,6^ allowing for steroid-free remission, mucosal and histological healing, and reductions in hospitalisations and surgeries.^6^ However, some limitations of biologics, including the risk for immunogenicity, high rates of primary non-response or secondary loss of response, safety concerns, and the requirement of parenteral administration, have yet to be overcome.^5–9^

Small molecule therapies provide advantages, including oral administration, little to no risk of immunogenicity, and the potential for rapidity of onset.^5,6^ The Janus kinase [JAK] inhibitors tofacitinib, upadacitinib, and filgotinib are effective oral small molecule therapies approved for the treatment of UC in multiple countries.^9–14^ However, the US Food and Drug Administration issued a black box warning for tofacitinib and upadacitinib due to an increased risk of serious infections, mortality, malignancy, major cardiovascular events, and thrombosis, and relegated the use of these therapies to patients who have failed TNF inhibitors.^10,12^ Recently, the European Medicines Agency has shared such concerns and added warnings and restrictions to the summary of product characteristics for both tofacitinib and upadacitinib.^11,13^ Filgotinib has not been approved by the US Food and Drug Administration, but the European Medicines Agency did not relegate the use of filgotinib to following TNF inhibitors and instead approved its use after conventional or biologic therapies.^14^ Therefore, there remains a need for novel oral small molecule therapies, particularly options that can be used after aminosalicylates before initiating other advanced therapies.

Ozanimod is an oral small molecule sphingosine 1-phosphate [S1P] receptor modulator.^15^ It is the first S1P receptor modulator approved in multiple countries for the treatment of moderately to severely active UC and is also approved in multiple countries for relapsing forms of multiple sclerosis [MS].^16,17^ The objective of this review is to provide guidance for ozanimod use for the treatment of UC based on the prescribing information, clinical trial and real-world data, and the authors’ clinical experiences.

## 2. Mechanism of Action of Ozanimod

**Figure 1.**
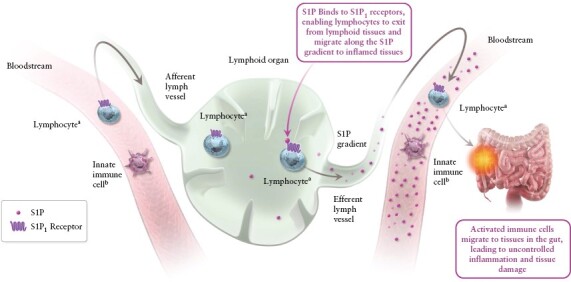
Role of S1P signalling in lymphocyte trafficking and inflammation. S1P molecules bind to S1P_1_ receptors, enabling lymphocytes to migrate from lymphoid tissues to inflamed tissues along the S1P gradient.^3,18,19^ S1P molecules also recruit activated immune cells and inflammatory markers to tissues in the gut, leading to uncontrolled inflammation and tissue damage.^3^ Although S1P molecules are prominent throughout the bloodstream, this is not depicted in the bloodstream on the left for simplicity in this conceptual illustration. ^a^Including T cells and B cells. ^b^Innate immune cells, which are responsible for antigen presentation and immunosurveillance, include macrophages, monocytes, and natural killer cells, among others. S1P, sphingosine 1-phosphate.

The pathogenesis of IBD is thought to involve the migration of lymphocytes from lymphoid tissues to the intestines, where they promote inflammation.^3,6^ S1P signalling is involved in lymphocyte trafficking and plays a role in inflammation.^3,18,19^ S1P binds to S1P_1_ receptors on lymphocytes, enabling them to migrate from lymphoid tissues into the circulation and to sites of inflammation [Figure 1].^3,18,19^ At sites of inflammation, S1P is involved in the recruitment of immune cells, which further exacerbates the inflammatory process.^3^ The enzymatic pathways that control S1P levels are dysregulated in the intestinal tissue of patients with IBD, suggesting that intestinal S1P levels are altered in IBD.^20,21^ Ozanimod is an S1P receptor modulator that binds with high affinity to the S1P_1_ and S1P_5_ receptor subtypes, leading to S1P_1_ receptor internalisation and thus reducing the capacity of lymphocytes to egress from lymphoid tissue.^15^ Ozanimod and its major active metabolites do not bind to the S1P_2–4_ receptors *in vitro*, limiting some of the off-target effects that are associated with non-selective S1P receptor modulators.^15,22–24^

## 3. Pharmacokinetics of Ozanimod

Ozanimod is metabolised into two major active metabolites [ie, CC112273 and CC1084037] and several other minor active metabolites, all with similar selectivity for S1P_1_ and S1P_5_.^17^ Approximately 94% of total circulating active drug is made up of ozanimod [6%], CC112273 [73%], and CC1084037 [15%]. The mean half-life of ozanimod is approximately 21 h and the mean half-life of CC112273 and CC1084037 is approximately 11 days. Given the relatively long half-life of the major active metabolites, some effects of ozanimod may continue after treatment discontinuation and a washout period of up to 3 months after discontinuing ozanimod is recommended in certain situations [eg, planning a pregnancy, planning to initiate an immunosuppressant or receipt of a live attenuated vaccine], and patients should be monitored for infections for up to 3 months after ozanimod is stopped, as discussed in the following sections of this review.^17^

## 4. Overview of the Efficacy and Safety Results from the Phase 3 True North Study

The efficacy of ozanimod 0.92 mg [equivalent to ozanimod HCl 1 mg] in patients with moderately to severely active UC was demonstrated in True North, a 52-week, randomised, double-blind, placebo-controlled phase 3 trial.^25^ Significantly more patients achieved clinical remission with ozanimod [18.4%] than placebo [6%] after 10 weeks of induction [*p* <0.001]; significantly more patients also achieved the secondary endpoints with ozanimod compared with placebo, including clinical response [47.8% vs 25.9%; *p* <0.001] and mucosal healing [defined as both endoscopic and histological response; 12.6% vs 3.7%; *p* <0.001]. After an additional 42 weeks of maintenance treatment among responders to ozanimod at Week 10, the primary endpoint of clinical remission was achieved by significantly more patients who continued ozanimod treatment [37%] than by those who were re-randomised to placebo [18.5%; *p* <0.001]; significantly more patients also achieved the secondary endpoints of clinical response [60% vs 41%; *p* <0.001], mucosal healing [29.6% vs 14.1%; *p* <0.001], and corticosteroid-free remission [31.7% vs 16.7%; *p* <0.001].

Ozanimod 0.92 mg was well tolerated in the True North trial.^25^ The occurrence of treatment-emergent adverse events [TEAEs] was similar between ozanimod and placebo during the induction period [40% and 38%, respectively], but higher with ozanimod than placebo during the maintenance period [49% and 37%, respectively]. The occurrence of serious TEAEs [4–6%] and TEAEs leading to treatment discontinuation [1–4%] with ozanimod was low. Adverse events of special interest [AESIs], based on prior association with S1P receptor modulation, were also assessed. Cardiac events were mitigated with gradual dose escalation, with bradycardia occurring in five patients [0.6%] on ozanimod during the induction period; only one of these patients experienced symptomatic bradycardia after the first dose, which resolved within 5 h and did not require treatment or extended monitoring. The occurrence of cancer [≤0.9%; included two cases of basal cell carcinoma, two cases of colorectal cancer, and one case of breast cancer], serious infection [≤1.6%; included appendicitis, gastroenteritis, nasopharyngitis, otitis externa, pyelonephritis, and vestibular neuronitis], and macular oedema [≤0.4%] were low with ozanimod in the induction and maintenance periods. No patients met the criteria for Hy’s law to suggest drug-induced liver injury, none had severe liver injury, and abnormal liver function tests infrequently led to treatment discontinuation [0.4% each for the induction and maintenance period] in patients receiving ozanimod. These results are overall consistent with safety findings reported in phase 3 trials of ozanimod for MS.^26,27^

These data have been supported by real-world data from an observational study of 30 patients with IBD who initiated ozanimod therapy at a single centre.^28^ At Week 10, 18 of 21 patients with clinically active UC had follow-up data available, 44% [8/18] had achieved clinical response and 33% [6/18] were in clinical remission, and 33% [6/18] achieved corticosteroid-free remission. AEs occurred in 26% of patients and included one case each of acute gastroenteritis, transient nausea, mild liver enzyme derangement, and transient chest pain; none required treatment discontinuation. There were also two cases of fatigue, one of which led to treatment discontinuation. Additionally, one patient with a history of hypertension developed a hypertensive urgency and discontinued ozanimod. There were no cases of symptomatic bradycardia, infections, or deaths.

## 5. Guide to Using Ozanimod for the Treatment of UC

### 5.1. Patient selection and suitability

Ozanimod is indicated for patients with moderately to severely active UC and is contraindicated for patients with certain conditions or taking certain medications [Table 1].^16,17^

**Table 1.** Patient selection, assessments and considerations prior to ozanimod initiation, and monitoring during ozanimod treatment.

Patient selection	
Indication: adults with moderately to severely active UC^16,17^	
The EU SmPC additionally specifies that patients should have had an inadequate response, lost response, or been intolerant to either conventional therapy or a biologic agent	
Contraindications^16,17^ • Myocardial infarction, unstable angina, stroke, transient ischaemic attack, decompensated heart failure requiring hospitalisation, or New York Heart Association class III/IV heart failure in the past 6 months • Mobitz type II second-degree or third-degree AV block, sick sinus syndrome, or sinoatrial block, unless the patient has a functioning pacemaker • Severe untreated sleep apnoea • Concomitant use of an MAO inhibitor [eg, selegiline, phenelzine, linezolid]	Additional contraindications per the EU SmPC^16^ • Immunodeficient state • Severe active infection or active chronic infection [eg, hepatitis, tuberculosis] • Active malignancy • Severe hepatic impairment [Child-Pugh class C] • Current pregnancy or lack of effective contraception use
**Preinitiation evaluation** ^16,17^	
Baseline examinations and laboratory tests	Other considerations
Complete blood count within past 6 months or after discontinuation of prior UC therapy ECG Liver transaminase and bilirubin levels within the past 6 months Ophthalmic examination of the fundus [including the macula] if patient has a history of diabetes, uveitis, or macular oedema VZV antibody test in patients without history of chickenpox or confirmed vaccination	Determine if patient is taking or has a history of taking antineoplastic, non-corticosteroid immunosuppressive, or immune-modulating therapies Determine if patient is taking drugs that could slow heart rate or AV conduction Determine if patient requires VZV vaccination or any other live attenuated vaccines Determine if patient has an active infection Determine if patient is pregnant or plans to become pregnant
**Monitoring during treatment**	
Blood pressure: monitor regularly during treatment^16,17^; per author recommendation, patients with pre-existing hypertension should monitor weekly at home for the first month; in all patients with or without hypertension, monitor 3 months after treatment initiation and then every 6 months thereafter Infection: monitor during treatment and for up to 3 months after treatment discontinuation^16,17^ ALC: monitor periodically^16^; the authors recommend assessing every 3 months Ophthalmic^16,17^ Monitor for changes in vision or symptoms of macular oedema [eg, blurriness, blind spot, or shadows in center of vision, sensitivity to light, unusually coloured vision] Patients with a history of diabetes, uveitis, or macular oedema should have regular ophthalmic examinations Hepatic Assess liver transaminase and bilirubin levels at 1, 3, 6, 9, and 12 months after treatment initiation and then periodically^16^; the authors recommend assessing every 3 months after the first year Monitor for symptoms of hepatic dysfunction [eg, unexplained nausea, vomiting, abdominal pain, fatigue, anorexia, jaundice, dark urine]^16,17^ Pulmonary: assess respiratory function if clinically indicated [eg, obstructive sleep apnoea, dyspnoea]^17^	

ALC, absolute lymphocyte count; AV, atrioventricular; ECG, electrocardiogram; MAO, monoamine oxidase; SmPC, summary of product characteristics; UC, ulcerative colitis, VZV, varicella zoster virus.

### 5.2. Screening assessments, considerations, and patient education prior to ozanimod treatment initiation

Table 1 provides a list of examinations, laboratory tests, and considerations that are recommended before initiating ozanimod treatment per the US prescribing information and EU summary of product characteristics. Supplementary Table 1 provides additional details regarding screening assessments, considerations, and patient education prior to ozanimod initiation. This information is summarised in the following paragraphs and supported by clinical trial and real-world data and author experience.

S1P receptor modulators as a class may result in transient reductions in heart rate and atrioventricular conduction due to S1P receptor binding in the heart.^29–31^ Tachyphylaxis to this effect occurs rapidly and is mitigated with the upward titration of dosing that is built into the ozanimod starter pack. Ozanimod’s lack of affinity for S1P_3_ receptors is relevant to its cardiovascular safety profile, as modulation of this receptor specifically has been shown to be associated with cardiac-conduction abnormalities.^15^ The ozanimod prescribing information recommends an electrocardiogram prior to ozanimod initiation to determine the presence of any pre-existing cardiac conduction abnormalities. Ozanimod is contraindicated for patients who have Mobitz type II second-degree or third-degree atrioventricular block, sick sinus syndrome, or sinoatrial block, unless the patient has a functioning pacemaker; consultation with a cardiologist is recommended for patients taking certain drugs that may have additive cardiac effects [eg, Class Ia or III anti-arrhythmic drugs, QTc-prolonging drugs, calcium channel blockers, beta-blockers] or with certain other cardiac conditions that are not contraindicated [Supplementary Table 1].^16,17^ Of note, real-world data in patients with IBD taking ozanimod showed that concomitant use of beta-blockers [in two patients] and QTc-prolonging drugs [in four patients] were not associated with any AEs.^28^

Although there is no clinical evidence of an interaction between ozanimod and tyramine, the US prescribing information advises that patients should be counselled about consuming foods or drinks containing high amounts of tyramine [ie, more than 150 mg]. This recommendation stems from the potential of an interaction that could cause severe hypertension, based on *in vitro* findings that ozanimod inhibits monoamine oxidase-B, an enzyme that provides protection from tyramine.^17^ Some foods, such as those that are aged, fermented, cured, smoked, and pickled, are high in tyramine. For example, soy sauce, aged cheddar cheese, and some alcoholic beverages [eg, unpasteurized craft beer, spontaneously fermented beer, wine] may have high levels of tyramine,^32,33^ but moderate consumption of these foods/drinks with ozanimod should be safe. It is the authors’ opinion that it is highly unlikely that a patient would ingest tyramine in a large enough amount to induce an interaction with ozanimod.

**Figure 2.**
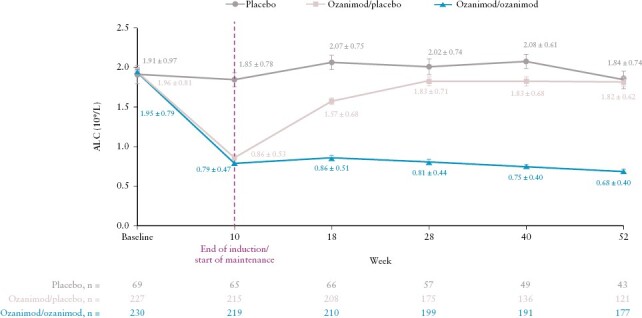
Mean ALC over time in the induction and maintenance periods of the phase 3 True North trial. Error bars denote standard error. ALC, absolute lymphocyte count.

By its mechanism of action, ozanimod has an immunosuppressive effect [ie, inflammatory-relevant lymphocyte sequestration in lymphoid tissues] that may increase susceptibility to infections.^16,17^ Physicians and patients should be made aware that reduction of absolute lymphocyte count [ALC] is an expected pharmacodynamic effect of ozanimod.^17,31^ Post hoc analyses from the phase 3 True North trial demonstrated a mean reduction from baseline in ALC of 41–45% [from a mean of ~1.95 to 0.79–0.86 × 10^9^/L] after 10 weeks of ozanimod treatment in patients with UC; these reductions were maintained for 42 additional weeks in patients who continued ozanimod treatment, but ALC recovered within 8 weeks and returned to pre-treatment levels within 18 weeks in patients who discontinued ozanimod [Figure 2].^34,35^ Real-world data also demonstrated ALC reductions with ozanimod, with a prospective, observational study of patients with IBD showing a mean reduction in ALC of 67% in 13 patients with baseline and follow-up ALC measures.^28^ Analyses from combined UC and MS trials reported a mean reduction in ALC of 45% from baseline at 3 months. After discontinuing ozanimod, the median time for peripheral blood lymphocytes to return to normal was 30 days, with approximately 80% to 90% of patients achieving levels within the normal range within 3 months. Immunosuppressants should be avoided or used with caution during treatment and up to 4 weeks after the last dose of ozanimod.^17^ A baseline complete blood count, including lymphocyte count, should be obtained prior to ozanimod initiation.^16,17^ There is no specific guidance on lymphocyte counts considered unsuitable for ozanimod treatment initiation, but the EU summary of product characteristics does recommend discontinuing treatment when ALC is <0.2 × 10^9^/L.^16^

Before initiating ozanimod, it is important to consider active infections, therapies with the potential for additive immunosuppressive effects with ozanimod, and vaccination requirements.^16,17^ Ozanimod initiation should be delayed in patients with an active infection and those without confirmed varicella zoster virus [VZV] antibodies; VZV vaccination is recommended for antibody-negative patients without a health care professional-confirmed history of chickenpox or documentation of a full course of VZV vaccination, preferably 1 month before initiating ozanimod, if a delay in treatment initiation is feasible based on the clinical condition of the patient.^16,17^ It is worth noting that VZV vaccination is widely recommended in UC patients over the age of 18 upon diagnosis or upon initiation of any immunosuppressive therapy, so many patients will likely have already received the vaccination. Post hoc analyses of phase 3 clinical trial data demonstrated that the risk of VZV with ozanimod in patients with UC and MS with VZV immunity was low [≤1%]; no cases resulted in treatment discontinuation and all cases were successfully treated with oral antivirals.^36^ In addition, whereas live attenuated vaccines are not recommended during ozanimod treatment,^16,17^ non-replicating live vaccines [eg, monkeypox vaccine] may be acceptable, based on the authors’ experience with allowing such vaccines during treatment with ozanimod and other immunosuppressive therapies. There is no official guidance regarding severe acute respiratory syndrome coronavirus 2 [SARS-CoV-2] vaccination, but post hoc analyses of clinical trial data showed that patients with MS who were treated with ozanimod during an open-label extension [OLE] developed serological response to SARS-CoV-2 vaccines, with 100% seroconversion after mRNA vaccination.^37^ However, some patients developed lower antibody concentrations and may benefit from booster doses.

Elevations of aminotransferases may occur with ozanimod, so liver enzymes and function should be assessed before initiating treatment.^16,17^ Ozanimod treatment is not recommended in patients with severe hepatic impairment [ie, Child-Pugh class C].^16,17^ Patients with abnormal liver function or persisting elevations of aspartate aminotransferase or alanine aminotransferase of two or more times the upper limit of normal [ULN] or direct bilirubin of one and a half times times the ULN were excluded from the phase 3 True North trial of ozanimod for UC.^25^ Of note, post hoc analyses of phase 1–3 clinical trial data demonstrated no impact of liver enzymes or bilirubin levels on ozanimod pharmacokinetics in patients with UC or MS.^38^

S1P receptor modulators have been associated with increased risk for macular oedema, especially in patients with pre-existing risk factors.^16,17^ Patients with a history of uveitis, macular oedema, or diabetes may use ozanimod, but an ophthalmic evaluation should be performed before treatment initiation to minimise the risk of macular oedema.^16,17^ Ophthalmic examinations should already be performed regularly in patients in high-risk groups, regardless of potential ozanimod treatment initiation.

An increased risk of cutaneous malignancies has been reported with some S1P receptor modulators.^17^ There are no official guidance on screening assessments related to malignancies. However, the EU summary of product characteristics outlines certain risks [ie, exposure to sunlight and concomitant phototherapy or photochemotherapy] that should be considered and recommends that ozanimod should not be initiated in patients with active malignancies.^16^ Ozanimod clinical trial data demonstrated a low incidence of malignancy [~1%] with long-term treatment in patients with UC for up to 142 weeks in the phase 3 True North OLE and in pooled phase 2 and phase 3 UC trials examining ozanimod over 2196 patient-years of exposure.^39,40^ Similar incidence [ie, any malignancy, 1.2%; cutaneous malignancy, 0.4%] was reported in patients with MS with up to 5 years of ozanimod treatment in an OLE that enrolled patients from phase 1–3 trials.^41^

Ozanimod may affect pulmonary function, as small reversible reductions in forced expiratory volume and forced vital capacity were observed with ozanimod in the UC and MS clinical trials.^16,17^ There is no official guidance on screening assessments related to the respiratory system, but the EU summary of product characteristics recommends that ozanimod should be used with caution in patients with severe respiratory disease, pulmonary fibrosis, or chronic obstructive pulmonary disease.^16^ In addition, ozanimod is contraindicated in patients with severe, untreated sleep apnoea per the US prescribing information.^17^

Data on the use of ozanimod during pregnancy are limited.^16,17^ Although some animal data suggested that ozanimod may affect foetal development,^16,17^ post hoc analyses of 4131 clinical trial participants receiving ozanimod found no evidence of increased adverse pregnancy outcomes among 56 women with UC or MS exposed to ozanimod during early pregnancy.^42^ Additionally, congenital anomalies and spontaneous early loss in partners of treated patients occurred at similar rates as in the general population. However, additional data are needed to further evaluate risks associated with ozanimod use during pregnancy or in partners of people who become pregnant. Notably, a pregnancy exposure registry currently enrolling women with MS is monitoring pregnancy outcomes in those exposed to ozanimod during pregnancy,^17^ and a pregnancy exposure registry for patients with UC is planned. People of childbearing potential should be counselled on the possible risk to the fetus, the use of contraception during treatment and for 3 months after stopping ozanimod, and how to proceed if pregnancy is planned or occurs while receiving ozanimod [ie, stop ozanimod 3 months before planning a pregnancy and discontinue treatment if pregnancy occurs during treatment].^16,17^

The efficacy and safety of ozanimod in elderly patients should be considered. Post hoc analyses of phase 3 clinical trial data from the True North study showed that ozanimod is safe and efficacious in patients with UC aged ≥60 years, although the sample size was small.^43^ Even so, elderly patients should be monitored, particularly for adverse hepatic and cardiac reactions, because of the greater frequency of hepatic and cardiac abnormalities in that population.^17^ Specifically, clinicians should check hepatic enzyme levels approximately once every 3 months and should instruct patients to report any cardiac-related symptoms. Studies found no clinically significant differences in the pharmacokinetics of ozanimod based on age in patients with UC and MS, indicating that dose adjustments are not necessary.^16,17,38^

In addition to potential cardiac and immunosuppressive drug interactions discussed in the preceding paragraphs, other potential drug interactions should be considered. Studies have demonstrated that the major active metabolites of ozanimod inhibit monoamine oxidase-B *in vitro*.^17,44^ Monoamine oxidase-B metabolises neurotransmitters, such as serotonin and norepinephrine, and inhibition of monoamine oxidase-B can lead to accumulation of these neurotransmitters.^44^ Consequently, concomitant use of medications that increase serotonin and norepinephrine could lead to hypertensive crisis. Per the US prescribing information, ozanimod use is contraindicated in patients taking monoamine oxidase inhibitors.^17^ In addition, the US prescribing information does not recommend co-administration of other medications that increase serotonin and norepinephrine (eg, opioids, selective serotonin reuptake inhibitors [SSRIs], serotonin and norepinephrine reuptake inhibitors [SNRIs], tricyclics), but advises that patients should be monitored for hypertension if concomitant use occurs.^17^ However, it is important to consider that post hoc analyses of clinical trial data demonstrated no evidence of increased incidence of hypertension with co-administration of ozanimod and SSRIs or SNRIs in patients with UC or MS; there was also no evidence of increased incidence of other serotonin syndrome-related TEAEs with concomitant use.^45^ This aligns with the authors’ clinical observations of very few issues with ozanimod treatment in patients receiving SSRIs or SNRIs, and with real-world data in patients with IBD reporting that no AEs were attributed to interactions with concomitant SSRI use in five patients or with concomitant tricyclic antidepressant use in another patient.^28^ Therefore, whereas there is not yet enough clinical evidence to conclude that ozanimod can be safely used in patients using these medications in clinical practice, the existing evidence suggests that the benefits of concomitant administration may outweigh the risks. In addition to drugs that increase norepinephrine [eg, SNRIs], consuming foods or drinks containing large amounts of tyramine may cause release of norepinephrine, which could result in a rise in blood pressure. The findings of no increased incidence of hypertension with concomitant use of SNRIs support the lack of clinical evidence of an interaction between ozanimod and tyramine, as discussed previously in this section.

The co-administration of ozanimod with strong CYP2C8 inhibitors may increase the exposure of active metabolites of ozanimod, potentially leading to increased risk of adverse reactions to ozanimod.^16,17^ The co-administration of ozanimod with strong CYP2C8 inducers may decrease the exposure of active metabolites of ozanimod, potentially leading to reduced ozanimod efficacy. Therefore, co-administration is not recommended.

See Table 2 for a summary of drugs that may interact with ozanimod. The authors would like to note that many of these potential interactions have not been studied thoroughly and thus require further exploration. The management of drug interactions in patients with comorbidities who require concomitant use of medications that potentially interact with ozanimod may require collaboration between general internists and gastroenterologists. It is generally recommended that prescribers review the patient’s medication list for drug interactions with ozanimod through any of the available online databases, or check with their pharmacist. Patients should also be counselled to inform their physician of any new prescribed medications, to allow for a review of potential drug-drug interactions.

**Table 2.** Potential drug-drug interactions with concomitant ozanimod treatment.^16,17^

Drug class	Drug examples	Potential interaction
Antineoplastic, non-corticosteroid immunosuppressive, or immune-modulating therapies^a^	Ciclosporin, tacrolimus, sirolimus, methotrexate, alemtuzumab, beta interferon, glatiramer acetate, azathioprine, 6-mercaptopurine	Additive immunosuppressive effect with concomitant ozanimod use
Anti-arrhythmic drugs, QT-prolonging drugs, or drugs that may decrease heart rate	Quinidine, procainamide, disopyramide [Class Ia anti-arrhythmic]; amiodarone, sotalol [Class III anti-arrhythmic]	Additive effect on heart rate with concomitant ozanimod use Anti-arrhythmic drugs have been associated with Torsades de Pointes in patients with bradycardia
Beta blockers	Propranolol, metoprolol, carvedilol, atenolol	Additive effect on heart rate if used in combination with a calcium channel blocker and ozanimod
Calcium channel blockers	Verapamil, diltiazem, amlodipine, felodipine, nifedipine	Additive effect on heart rate if used in combination with a beta blocker and ozanimod
Opioid drugs	Meperidine, methadone, tramadol, oxycodone, hydrocodone, morphine, codeine, fentanyl	Serious adverse reactions, including hypertensive crisis, with concomitant ozanimod use due to potential MAO inhibition by ozanimod metabolites
Serotonergic/adrenergic drugs	Citalopram, escitalopram, fluoxetine, paroxetine, sertraline [SSRI]; desvenlafaxine, duloxetine, levomilnacipran, venlafaxine [SNRI]; amitriptyline, amoxapine, desipramine, doxepin, imipramine, nortriptyline, protriptyline, trimipramine [tricyclic]	Serious adverse reactions, including hypertensive crisis, with concomitant ozanimod use due to potential MAO inhibition by ozanimod metabolites
Sympathomimetic drugs^b^	Pseudoephedrine, phenylephrine, amphetamines, midodrine, clonidine, albuterol, salmeterol, terbutaline	Serious adverse reactions, including hypertensive crisis, with concomitant ozanimod use due to potential MAO inhibition by ozanimod metabolites
MAO inhibitors	Selegiline, phenelzine, linezolid, isocarboxazid, tranylcypromine	Reduced exposure to active metabolites of ozanimod with concomitant ozanimod use, which may decrease the efficacy of ozanimod Risk of hypertensive crisis with concomitant ozanimod use due to potential MAO inhibition by ozanimod metabolites
CYP2C8 inhibitors	Gemfibrozil, clopidogrel	Increased exposure to active metabolites of ozanimod with concomitant ozanimod use, which may increase the risk of adverse reactions to ozanimod
CYP2C8 inducers	Rifampin	Reduced exposure to active metabolites of ozanimod with concomitant ozanimod use, which may decrease the efficacy of ozanimod

AV, atrioventricular; MAO, monoamine oxidase; SNRI, serotonin and norepinephrine reuptake inhibitor; SSRI, selective serotonin reuptake inhibitor; VZV, varicella zoster virus.

^a^Ozanimod has only been studied with ciclosporin, which had no pharmacokinetic interaction.

^b^Ozanimod [which inhibits MAO-B *in vitro*] has only been studied with pseudoephedrine, which did not result in any potentiated effects on blood pressure, but hypertensive crisis has been reported with concomitant use of other MAO inhibitors and sympathomimetic drugs.

### 5.3. Washout of prior therapies

The ozanimod US prescribing information and EU summary of product characteristics provides some guidance for switching to ozanimod from immunosuppressive treatments, recommending that the half-lives and modes of action of those treatments be considered, to avoid additive immune effects while also minimising risk of disease relapse.^16,17^ In the phase 3 True North trial of ozanimod, patients could be excluded based on recent use of some UC therapies including treatment with a biologic agent within 8 weeks or five elimination half-lives [whichever was less] or an investigational agent within five elimination half-lives prior to randomisation and treatment with tofacitinib within 2 weeks of screening.^25^ However, washout of previous UC therapies is typically an academic concept used in clinical trials which is not necessary in practice, as ozanimod has a unique mechanism of action compared with other existing UC therapies. In clinical practice in some countries, medication switches are typically initiated as soon as insurance approval is received, without any washout period. Of note, washout of prior therapies is not necessary with azathioprine treatment as long as differential blood counts are normal. Therefore, it is the authors’ opinion that washout of previous UC therapies before initiating ozanimod treatment is not a concern unless the patient has abnormal differential blood counts, such as significant lymphopenia [eg, ALC less than 0.5 × 10^9^/L]. A complete blood count is recommended for all patients prior to ozanimod initiation regardless of prior therapy use, per the US prescribing information and EU summary of product characteristics.^16,17^

### 5.4. Ozanimod treatment initiation and continued dosing

Binding to the S1P_3_ receptor subtype is believed to mediate chronotropic cardiac effects observed with non-selective S1P receptor modulators.^29^ Although ozanimod is a selective S1P receptor modulator with very low affinity for the S1P_3_ receptor subtype, bradycardia may still occur due to its affinity for the S1P_1_ receptor subtype.^15,29^ However, the risk of bradycardia can be mitigated with gradual dose titration.^29,31^ Therefore, it is recommended to initiate ozanimod as follows: ozanimod 0.23 mg [equivalent to ozanimod HCl 0.25 mg] once daily on Days 1–4, ozanimod 0.46 mg [equivalent to ozanimod HCl 0.5 mg] once daily on Days 5–7, and ozanimod 0.92 mg once daily from Day 8 and thereafter.^16,17^

First-dose cardiac monitoring is not required per the US prescribing information.^17^ However, the EU summary of product characteristics recommends heart rate and blood pressure monitoring for 6 h after the first dose [with an electrocardiogram before and after the 6-h period] for patients with certain pre-existing cardiac conditions [ie, resting heart rate <55 bpm, Mobitz type I, second-degree, atrioventricular block, or history of myocardial infarction or heart failure].^16^ Additional monitoring is recommended if, at Hour 6, heart rate is <45 bpm or at its lowest measured value since first dose, new-onset, second-degree or higher, atrioventricular block occurs, or QTc interval is ≥500 ms. The authors would like to note that patients with comorbidities requiring first-dose monitoring will likely account for a very small proportion of patients with UC and may not be best suited for treatment with ozanimod.

Post hoc analyses of phases 2 and 3 clinical trial data confirmed that the ozanimod dose titration schema mitigates first-dose cardiac effects in patients with UC and MS; transient and minimal reductions in heart rate occurred with the first dose of ozanimod, and there were few [<1%] first-dose, cardiac AEs with ozanimod in patients with or without a history of cardiac disorders.^46^ Additional post hoc analyses of phase 3 clinical trial data demonstrated a low incidence of cardiac AEs [≤2.2%] and no increased risk of thromboembolic events or major adverse cardiac events [eg, cardiovascular death, myocardial infarction, stroke] with up to 1 year of ozanimod treatment in patients with UC; discontinuations due to cardiac AEs were low [<1%] and there were no cardiovascular-related deaths.^47,48^ A similar incidence of cardiac AEs [2.8%] was reported in patients with MS with up to 5 years of ozanimod treatment in an OLE that enrolled patients from phase 1–3 trials.^41^

The EU summary of product characteristics recommends dose adjustment in patients with mild or moderate chronic hepatic impairment [Child-Pugh Class A or B], instructing these patients to complete the 7-day dose titration regimen and then take the final dose of 0.92 mg once every other day thereafter.^16^ There are no dose adjustments required in the US prescribing information.^17^

The 7-day dose titration regimen is recommended to be repeated when a dose is missed ≥1 times during the first 2 weeks of treatment, for more than 7 consecutive days during the third and fourth weeks of treatment, or for more than 14 consecutive days after the first month of treatment.^16,17^ Otherwise, treatment should be continued as planned.

### 5.5. Expected time to efficacy with ozanimod

Post hoc analyses from the phase 3 True North trial demonstrated an improvement in rectal bleeding and stool frequency subscores as early as Weeks 2 and 5, respectively, compared with placebo.^49^ Similarly, significantly higher rates of symptomatic response and remission were observed for ozanimod compared with placebo as early as 2 weeks and 5 weeks, respectively.^50^ Real-world data also demonstrated efficacy as early as 2 weeks, with 7/16 patients [44%] achieving clinical response and 3/16 [19%] clinical remission; one patient [6%] achieved corticosteroid-free remission following 2 weeks of ozanimod.^28^

Additional post hoc analyses from True North demonstrated that approximately half of the patients who did not respond to ozanimod within 10 weeks of treatment initiation were able to achieve symptomatic clinical response with an additional 5 to 10 weeks of extended induction treatment.^51^ Rates of symptomatic clinical response with up to 10 weeks of ozanimod extended induction were similar in biologic-naive and biologic-exposed patients.^52^ Further analyses showed that patients who required extended induction treatment were able to maintain symptomatic clinical response, as well as clinical, endoscopic, and histological efficacy, for up to 2 years, regardless of prior biologic exposure.^53–55^ Overall, these data indicate that extended induction treatment should be considered for patients who do not initially respond to ozanimod.

Overall, improvements were seen with ozanimod in the True North study earlier in biologic-naive patients compared with patients previously exposed to biologics.^25,50,56^ Post hoc analyses demonstrated greater efficacy of ozanimod in biologic-naive patients than biologic-exposed patients during the initial 10 weeks of induction therapy, but similar efficacy regardless of biologic exposure after an additional 42 weeks of therapy in patients who responded to induction therapy and entered the maintenance period.^56^ Similarly, patients who were naive to TNF inhibitors achieved significant improvements with ozanimod compared with placebo at the end of the induction and maintenance periods of True North; patients who had prior exposure to TNF inhibitors had numerically greater improvements compared with placebo after 10 weeks of ozanimod induction therapy, and these improvements were significant at the end of maintenance evaluation [Week 52].^25^ In an additional post hoc analysis, patients who were naive to TNF inhibitors achieved significantly higher rates of symptomatic response and remission compared with placebo 2 weeks and 4 weeks, respectively, after initiating ozanimod; these responses occurred later in patients with prior exposure to TNF inhibitors [4 weeks and 8 weeks, respectively].^50^ Overall, these data indicate that patients with prior biologic exposure may require additional time to respond to ozanimod treatment. However, it should be noted that this was not seen in a real-world study of 21 patients with clinically active UC, which reported no significant difference in response or remission rates between patients with or without prior exposure to other advanced therapies, including vedolizumab, after 10 weeks of ozanimod therapy.^28^

### 5.6. Safety monitoring during ozanimod treatment

Table 1 summarises monitoring requirements from the US prescribing information and EU summary of product characteristics or that are recommended by the authors. Supplementary Table 2 provides additional details regarding monitoring during ozanimod treatment. This information is summarised in the following paragraphs and supported by clinical trial data and author experience.

Blood pressure should be monitored during ozanimod treatment due to the potential for increased blood pressure.^16,17^ There is no official guidance on the timing or frequency of monitoring, but it is the authors’ opinion that patients with pre-existing hypertension should be instructed to monitor their blood pressure weekly for the first month of treatment; these patients should already have monitoring equipment available to them to manage hypertension, so weekly office visits are not needed. Regardless of hypertension history, blood pressure should be checked after 3 months of ozanimod treatment, when blood pressure changes were first observed in clinical trials.^17^ If no increases in blood pressure occur, monitoring should continue every 6 months at follow-up visits or if symptoms of hypertension occur. Blood pressure monitoring may be done by the gastroenterologist or in collaboration with a general internist. There are no other special considerations or additional monitoring requirements for patients with well-controlled hypertension, provided that the pre-initiation ECG was normal and does not require further attention, as described above, due to cardiac conduction abnormalities that could result from antihypertensive therapy. Of note, post hoc analyses of clinical trial data from the phase 3 True North trial demonstrated small increases in mean blood pressure which stabilised over time with ozanimod treatment for up to 1 year in patients with UC; hypertension occurred in <2% of patients receiving ozanimod and no cases resulted in treatment discontinuation.^47^ A slightly higher incidence of hypertension [7.5%] was reported in patients with MS with up to 5 years of ozanimod treatment in an OLE that enrolled patients from phase 1–3 trials.^41^

Patients should be instructed to promptly report symptoms of infection during and after ozanimod treatment.^16,17^ Clinical trial data demonstrated a low incidence of serious infections [≤2.7%] with long-term ozanimod treatment in patients with UC for up to 142 weeks in the phase 3 True North OLE and in pooled phase 2 and phase 3 UC trials examining ozanimod over 2196 patient-years of exposure.^39,40^ Similar incidence [2.8%] was reported in patients with MS with up to 5 years of ozanimod treatment in an OLE that enrolled patients from phase 1–3 trials.^41^

Post hoc analyses of clinical trial data showed that most ozanimod-treated patients with UC or MS who contracted COVID-19 had non-serious infections [89.8%], recovered [98.6%], and did not discontinue ozanimod treatment [99.5%]; most patients with serious COVID-19 fully recovered without sequelae.^57^ Therefore, ozanimod treatment can be continued during COVID-19 infection if warranted.

Ozanimod treatment should be interrupted or discontinued with certain infections [ie, serious infection, progressive multifocal leukoencephalopathy, cryptococcal meningitis, and posterior reversible encephalopathy].^16,17^ Progressive multifocal leukoencephalopathy [PML] has been reported with the S1P receptor modulator fingolimod in patients with MS, with an estimated global incidence rate of 4.75 per 100,000 patient-years.^58^ The incidence of PML appeared to increase with fingolimod treatment duration, plateauing at Year 5. To date, one case of PML has been reported with ozanimod in a 46 year-old female patient with a 15-year history of MS after approximately 4 years of ozanimod treatment; ozanimod was discontinued and the patient had a non-fatal outcome with neurological sequelae.^41^ This is the first known case of PML across over 16,000 patient-years of ozanimod exposure in patients with IBD and MS and across nearly 6500 patient-years of postmarketing exposure. Importantly, there have been no cases of PML reported with ozanimod in UC patients. There are no data on the risk of surgical complications, such as infections, with ozanimod. Therefore, health care providers should be cautious of infections after any surgical procedure is performed while a patient is receiving ozanimod.

Post hoc analyses from True North demonstrated that ALC change is a pharmacodynamic marker of ozanimod exposure, but changes in ALC were not associated with its efficacy or safety.^59^ Although ALC reduction is an expected pharmacodynamic effect of ozanimod, the EU summary of product characteristics recommends periodic ALC monitoring and treatment interruption if lymphopenia occurs [Table 1; Supplementary Table 2].^16^ Whereas there is no official guidance on frequency of monitoring, it is the authors’ opinion that ALC should be monitored every 3 months. Post hoc analyses of clinical trial data from the phase 3 True North trial demonstrated that <2% of patients with UC had ALC <0.2 × 10^9^/L during ozanimod treatment and that ALC returned to >0.2 × 10^9^/L when testing was repeated while remaining on ozanimod.^34,35^ No patients with UC discontinued ozanimod due to low ALC [<0.5 or <0.2 × 10^9^/L], and none had a serious or opportunistic infection with concurrent low ALC [<0.2 × 10^9^/L].

In addition to an ophthalmic evaluation prior to ozanimod initiation in patients with diabetes or history of uveitis or macular oedema, follow-up evaluations should be performed regularly during ozanimod treatment in these patients, or in any patient with changes in vision where macular oedema is suspected [eg, distorted contour and colour of objects, unequal size with monocular view].^16,17^ Clinical trial data demonstrated a low incidence of macular oedema [≤0.6%] with long-term ozanimod treatment in patients with UC for up to 142 weeks in the phase 3 True North OLE and in pooled phase 2 and phase 3 UC trials examining ozanimod over 2196 patient-years of exposure; most cases occurred in patients with pre-existing risk factors or comorbid conditions.^39,40^ Similar incidence [0.4%] was reported in patients with MS with up to 5 years of ozanimod treatment in an OLE that enrolled patients from phase 1–3 trials, with three of four confirmed cases occurring in patients with pre-existing risk factors or comorbid conditions.^41^

Liver enzymes and function should be assessed periodically during treatment (ie, after 1, 3, 6, 9, and 12 months on ozanimod [per the EU summary of product characteristics] and every 3 months thereafter [per author recommendation]) or assessed with symptoms of hepatic dysfunction. Treatment should be interrupted if transaminases are confirmed to be >5 times the ULN [per the EU summary of product characteristics] and discontinued in the event of liver injury.^16,17^ Post hoc analyses of clinical trial data from the phase 3 True North trial demonstrated that elevations of liver enzymes and bilirubin did occur but were asymptomatic, transient, and generally resolved within 3 months without treatment discontinuation in patients with UC who were treated with ozanimod for up to 1 year; no Hy’s law cases or severe drug-induced liver injury occurred.^60^ Similar findings were reported in patients with MS with up to 5 years of ozanimod treatment, in an OLE that enrolled patients from phase 1–3 trials.^41^

Respiratory function should be assessed if clinically indicated [eg, if the patient presents with obstructive sleep apnoea, new complaint of dyspnoea on exertion, or shortness of breath].^17^ Clinical trial data showed that pulmonary AEs were low [1%] and did not increase over time with 2196 patient-years of exposure.^39^

## 6. Placement of Ozanimod Within the Treatment Armamentarium for UC

**Figure 3.**
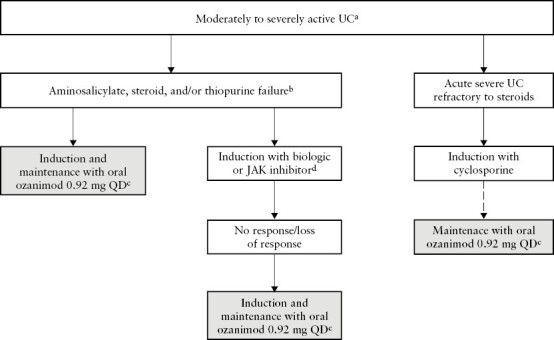
Proposed positioning of ozanimod within the UC treatment armamentarium. Solid arrows indicate a switch in therapy in patients without a response to the previous therapy. Dashed arrows indicate a switch in patients who achieved response to the previous therapy. ^a^US prescribing information states that ozanimod is indicated for adults with moderately to severely active UC. The EU summary of product characteristics additionally specifies that ozanimod is indicated for adults with moderately to severely active UC who have had an inadequate response, lost response, or been intolerant to either conventional therapy or a biologic agent. ^b^A typical patient requires >1 course of steroids in a year after starting an aminosalicylate or thiopurine. ^c^The 7-day dose titration regimen is required when ozanimod is initiated, regardless of whether it is being used as induction or maintenance therapy. ^d^Patients in remission on a biologic or JAK inhibitor who are inconvenienced by injections [biologics only] or worried about safety may switch to ozanimod for maintenance therapy. ^e^The transition from ciclosporin induction to ozanimod maintenance is recommended based on a case study reporting successful transition from ciclosporin to ozanimod in two patients who were hospitalised with acute severe UC refractory to steroids.^63^ JAK, Janus kinase; QD, once daily; UC, ulcerative colitis.

Society recommendations for the positioning of ozanimod within the treatment armamentarium for UC have yet to be published. Given that results from the phase 3 True North trial demonstrated that ozanimod is efficacious in both biologic-naive and biologic-exposed patients with UC,^25,56^ ozanimod could be positioned in several ways [Figure 3]. There is a clear lack of oral agents that fall between aminosalicylate or steroid failure and use of the first biologic therapy in the treatment algorithm. Evidence suggests that ozanimod may be best positioned before biologics, given the greater effect size of ozanimod in biologic-naive than biologic-exposed patients during induction therapy.^25,56^ Therefore, ozanimod could be used as a first-line therapy after aminosalicylate, steroid, and/or thiopurine failure. A typical patient is one who requires more than one course of steroids in a year after starting an aminosalicylate or thiopurine, but ozanimod may be used in any patient who does not respond or loses response to aminosalicylates, regardless of whether they required steroids. However, ozanimod could also be positioned after biologics in patients who fail or lose response to biologics, or in patients in remission with biologic therapy but who are inconvenienced by injections or worried about safety. Ozanimod could also be an option to maintain remission after induction with another advanced therapy [eg, ciclosporin, biologics, JAK inhibitors]. Acute, severe UC refractory to steroids is typically treated with the TNF inhibitor infliximab or the calcineurin inhibitor ciclosporin.^61,62^ A recent case study described the first report of the successful transition from ciclosporin induction therapy to ozanimod maintenance therapy in two patients who were hospitalised with acute, severe UC refractory to steroids^63^; the potential role of ozanimod maintenance after infliximab induction has not been investigated. Real-world data on transition from other advanced therapies to ozanimod in patients with moderately to severely active UC are also not yet available.

The risk profile of ozanimod compared with other UC therapies should be considered in the individual assessment of the benefit-risk ratio when making treatment decisions. Thiopurines are associated with treatment discontinuation because of an AE in up to one-third of patients,^64,65^ whereas AEs leading to ozanimod discontinuation were low [1–4%] in patients with UC, as observed in the phase 3 True North trial.^25^ Thiopurines are associated with an increased risk for non-melanoma skin cancer and lymphoma in patients with IBD; the risk of lymphoma increases after as little as 1 year of thiopurine exposure and the risk of non-melanoma skin cancer increases after 4 years of thiopurine exposure.^65–68^ Unlike ozanimod, thiopurines are also associated with a risk of pancreatitis and myelotoxicity.^65,67,68^ Further, thiopurines are not typically used as induction therapy, due to the relatively long onset of action of up to 3 months.^66,68,69^ TNF inhibitors are associated with immunogenicity, which can lead to loss of response; no evidence of immunogenicity was seen during the clinical trials of ozanimod. Biologic therapies have also been associated with infusion- or injection-related AEs^70^; this risk is circumvented by the oral route of administration of ozanimod.^16,17^ Some TNF inhibitors [ie, adalimumab and infliximab] and JAK inhibitors [ie, tofacitinib and upadacitinib] carry black-box warnings due to the risk of serious infections and malignancies.^10,12,71,72^ The risk of serious infections and malignancies was low with ozanimod in UC clinical trials,^39,40^ and ozanimod does not carry a black-box warning.^16,17^

## 7. Outlook on Ozanimod for the Treatment of Crohn’s Disease

In addition to UC, ozanimod has shown promise as a treatment for Crohn’s disease. In a 12-week, phase 2, uncontrolled trial, ozanimod 0.92 mg resulted in clinical, endoscopic, and histological improvements in patients with moderately to severely active Crohn’s disease.^73^ A phase 3 clinical trial programme further investigating ozanimod induction and maintenance therapy for moderately to severely active Crohn’s disease [YELLOWSTONE; NCT03440372, NCT03440385, and NCT03464097] is currently ongoing and expected to be completed in 2024. The positive, preliminary phase 2 findings suggest that ozanimod is likely to become an option for Crohn’s disease in addition to UC.

## 8. Conclusion

This review provides clinicians with a guide on how to educate patients and initiate ozanimod, and sets expectations regarding efficacy and safety during ozanimod treatment. The benefits and risks of ozanimod must be assessed for each patient. Those who may benefit most from ozanimod include young patients with moderately to severely active UC, patients who have previously received advanced therapies, patients who are naive to advanced therapies and are concerned about convenience or other features of those treatments, and patients with specific safety risks with other treatments. On the other hand, certain patients may be at higher risk with ozanimod treatment. In addition to patients for whom ozanimod is contraindicated [see Table 1], the following are some other potential risk factors to consider with ozanimod treatment: pre-existing cardiac conditions not included in the contraindications, high risk for macular oedema, concomitant use of cardiac or immunosuppressive medications, concomitant use of phototherapy or photochemotherapy, and respiratory disease.^16,17^

The nature and frequency of monitoring during ozanimod treatment should be adapted to the individual patient, based on their treatment-associated risk factors and AEs that occur during treatment. In this review, we have outlined the main risk factors to consider with ozanimod treatment. We have also provided guidance for routine monitoring and when to increase or change monitoring practices, based on the risk factors listed above and the occurrence of certain events [eg, liver enzyme elevation, lymphopenia, infection, and pregnancy]. Generally, the authors believe that gastroenterologists should collaborate with general internists for patients with risk factors and comorbidities that require special considerations or increased screening or monitoring with ozanimod use. This will be increasingly necessary with the aging of UC patients, which presents a challenge in managing the interaction between comorbidities, UC therapy, and UC-specific treatment approaches. Additional postmarketing and real-world data and continued experience with ozanimod use will provide further clarity regarding the patients most suitable for ozanimod treatment, the efficacy and safety of ozanimod, drug-drug interactions, and the positioning of ozanimod within the UC treatment landscape.

## Supplementary Material

jjad112_suppl_Supplementary_Table

## Data Availability

Bristol Myers Squibb policy on data sharing may be found at https://www.bms.com/researchers-and-partners/independent-research/data-sharing-request-process.html.
