# Supplementary material for: Clinician’s Guide to Using Ozanimod for the Treatment of Ulcerative Colitis
Source: J Crohns Colitis. 2023 Jul 12;17(12):2012–25. doi: 10.1093/ecco-jcc/jjad112 (PMC10798866; doi:10.1093/ecco-jcc/jjad112)
Supplement: jjad112_suppl_Supplementary_Table [file jjad112_suppl_supplementary_table.docx]

**SUPPLEMENTARY MATERIALS**

**Supplementary Table 1.** Screening assessments, considerations, and patient education prior to ozanimod initiation as outlined in the prescribing information16,17

| **Category** | **Assessment** | **Action** |
| --- | --- | --- |
| **Cardiovascular** | Perform an ECG  Determine if patient is taking any medications that could slow heart rate or AV conduction (eg, beta-blockers, calcium-channel blockers) | Consult cardiologist for the following patients:  • With significant QT prolongation (QTcF >450 msec in males and >470 msec in females in the US PI; QTc >500 msec in the EU SmPC)  • With risks for QT prolongation (only in the EU SmPC)  • Taking medications other than beta-blockers and calcium-channel blockers that may potentiate bradycardia (only in the EU SmPC)  • With arrhythmias requiring treatment with Class Ia (eg, quinidine, disopyramide) or Class III (eg, amiodarone, sotalol) antiarrhythmic drugs  • With ischemic heart disease, heart failure, history of cardiac arrest or myocardial infarction, cerebrovascular disease, and uncontrolled hypertension  • With severe untreated sleep apnea or history of recurrent syncope or symptomatic bradycardia (only in the EU SmPC)  • With a history of second-degree Mobitz type II and higher AV block, sick-sinus syndrome, or sinoatrial block  Consider potential additive effects; ozanimod is generally not recommended for patients taking both a beta-blocker and a calcium-channel blocker  Although there is no clinical evidence of an interaction between ozanimod and tyramine, the US PI advises that patients be counseled to avoid foods (eg, aged, fermented, cured, smoked, and pickled foods, such as aged cheese and pickled herring) and drinks (eg, spontaneously fermented beer, craft beers from microbreweries that are produced without a filtration or pasteurization process, draught beer) with large amounts (ie, >150 mg) of tyramine due to risk of severe hypertension resulting from a potential interaction between tyramine and ozanimod |
| **Immune status/infections** | Obtain complete blood count (including lymphocyte count) at baseline  Determine if patient has an active infection  Test for VZV antibodies in patients without a healthcare provider–confirmed history of chickenpox or without documentation of a full course of vaccination against VZV  Determine if patient requires any live attenuated vaccine  Determine if patient is taking or has recently taken antineoplastic, non–corticosteroid immunosuppressivec (ie, azathioprine, 6-mercaptopurine), and immune-modulating therapies) | No official guidance during screening, but ozanimod discontinuation is recommended when ALC is <0.2 x 10^9^/La; reinitiation of ozanimod may be considered when ALC returns above 0.5 x 10^9^/L (only in EU SmPC)  Delay initiation of ozanimod in patients with an active infection until the infection has resolved  Administer vaccination for antibody-negative patients ≥1 month before initiating ozanimod, if feasible, to delay treatment based on patient condition  Administer vaccination ≥1 month before initiating ozanimod and counsel patient to avoid live attenuated vaccines during ozanimod treatment and for up to 3 months after treatment discontinuationb due to risk of infection  Avoid these therapies or use caution with coadministration while considering potential additive immunosuppressive effects |
| **Hepatic** | Obtain transaminase and bilirubin levels at baseline | Ozanimod is not recommended in patients with hepatic impairment (ie, Child-Pugh class C)d |
| **Ophthalmic** | Perform an ophthalmic evaluation of the fundus, including the macula, in patients at high risk for macular edema (ie, patients with history of uveitis or macular edema and patients with diabetes) | Weigh the potential risks and benefits of ozanimod in patients with macular edema; counsel patients to get regular follow-up examinations during treatment |
| **Malignancy** | No official guidance on screening assessment | Caution patients against exposure to sunlight without protection and should not receive concomitant phototherapy with UV-B radiation or PUVA photochemotherapy (only in EU SmPC) |
| **Respiratory** | No official guidance on screening assessment | Use caution in patients with severe respiratory disease, pulmonary fibrosis, and chronic obstructive pulmonary disease (only in EU SmPC) |
| **Pregnancy** | Determine if female patient is pregnant or plans to become pregnant | Counsel people of childbearing potential on the possible risk to the fetus and advise them to use contraception to avoid pregnancy during ozanimod treatment and for up to 3 months after treatment discontinuationb  Counsel people of childbearing potential that ozanimod should be discontinued 3 months before planning a pregnancy or discontinued if a pregnancy occurs (only in EU SmPC) |
| **Age** | No official guidance on screening assessment in elderly patients | Monitor closely for adverse cardiac reactions (ie, instruct patients to report any symptoms) and hepatic reactions (ie, assess hepatic enzyme levels approximately once every 3 months) due to greater frequency of reduced cardiac and hepatic function |
| **Drug interactions** | Identify current medication use | The US PI does not recommend coadministration of SSRIs or SNRIs with ozanimod and recommends patient monitoring for hypertension if concomitant use occurs, but there is no evidence of adverse reactions with concomitant ozanimod use  Coadministration of strong CYP2C8 inhibitors or inducers with ozanimod is not recommended |

aThe phase 3 True North study of ozanimod for UC excluded patients if they had received previous treatment with lymphocyte-depleting therapies or had ALC <800/µL.25

bDue to the elimination time for ozanimod.

cConcomitant use of corticosteroids did not affect the safety and efficacy of ozanimod in clinical trials; however, long-term data are limited, so corticosteroids should also be coadministered with caution.

dThe phase 3 True North study of ozanimod for UC excluded patients with liver function impairment or persisting elevations of AST or ALT >2 times the ULN or direct bilirubin >1.5 times the ULN.25

ALC, absolute lymphocyte count; ALT, alanine aminotransferase; AST, aspartate aminotransferase; AV, atrioventricular; ECG, electrocardiogram; PI, prescribing information; SmPC, summary of product characteristics; SSRI, selective serotonin reuptake inhibitor; SNRI, serotonin and norepinephrine reuptake inhibitor; UC, ulcerative colitis; ULN, upper limit of normal; VZV, varicella zoster virus.

**Supplementary Table 2.** Monitoring during ozanimod treatment as outlined in the prescribing information16,17 or per author recommendation

| **Assessment** | **Action** |
| --- | --- |
| Per US PI and EU SmPC, assess BP regularly during treatment. BP monitoring may be done in collaboration with a general internist.  Per author recommendation, advise weekly at-home BP monitoring for the first month of ozanimod treatment in patients with preexisting hypertension. Monitor BP 3 months after ozanimod treatment initiation in all patients with or without hypertension; if no increases in BP occur, monitoring should continue every 6 months at follow-up visits or if symptoms of hypertension occur. | Initiate appropriate antihypertensive therapy if hypertension occurs per author recommendation |
| Per US PI and EU SmPC, monitor for infections during treatment and for up to 3 months after treatment discontinuationa | Per US PI and EU SmPC:  • Interrupt treatment if the patient develops a serious infection  • Interrupt treatment if patient develops signs/symptoms of PML or cryptococcal meningitis until diagnosis is excluded  • Discontinue treatment if patient is diagnosed with PML or if PRES is suspected  • Be cautious of additive immunosuppressant effects with recent or concomitant use of antineoplastic, immunosuppressive, or immune-modulating therapies |
| Per EU SmPC, monitor ALC periodically; the authors recommend assessing ALC every 3 months | Per EU SmPC, interrupt treatment if ALC is confirmed below 0.2 × 10^9^/L; reinitiate treatment when ALC returns above 0.5 × 10^9^/L |
| Per US PI and EU SmPC, monitor for changes in vision or symptoms of macular edema (eg, blurriness or shadows in center of vision, sensitivity to light, blind spot in center of vision, unusually colored vision) | Per US PI and EU SmPC, perform ophthalmic evaluation; discontinue ozanimod or weigh potential risks and benefits of continuing treatment in patients who develop macular edema |
| Per EU SmPC, assess liver transaminase and bilirubin levels at 1, 3, 6, 9, and 12 months after treatment initiation, and then periodically thereafter; the authors recommend assessing every 3 months after the first year  Per US PI and EU SmPC, monitor for symptoms of hepatic dysfunction (ie, unexplained nausea, vomiting, abdominal pain, fatigue, anorexia, jaundice, and/or dark urine) | Per EU SmPC, interrupt treatment if transaminases are confirmed to be >5 times the ULN and monitor levels frequently; reinitiate treatment when levels normalize  Per US PI and EU SmPC, discontinue treatment if liver injury occurs |
| Per US PI, assess respiratory function if clinically indicated (eg, obstructive sleep apnea, new complaint of dyspnea on exertion, shortness of breath) | Per US PI, perform spirometry if clinically indicated to assess FVC and FEV, and interrupt treatment if needed. There is insufficient information to determine if decreases in FEV_1_ or FVC could be progressive with continued use, or if changes are reversible after ozanimod discontinuation |

aDue to the elimination time for ozanimod.

ALC, absolute lymphocyte count; BP, blood pressure; FVC, forced vital capacity; FEV, forced expiratory volume; PI, prescribing information; PML, progressive multifocal leukoencephalopathy; PRES, posterior reversible encephalopathy; SmPC, summary of product characteristics; ULN, upper limit of normal.
